# Data on antibiograms and resistance genes of Enterobacterales isolated from ready-to-eat street food of Ambato, Ecuador

**DOI:** 10.12688/f1000research.117116.1

**Published:** 2022-06-17

**Authors:** Jessica Tubón, Gabriela Barragán-Fonseca, Liliana Lalaleo, William Calero-Cáceres

**Affiliations:** 1 UTA RAM One Health, Department of Food and Biotechnology Science and Engineering, Universidad Técnica de Ambato, Ambato, 180103, Ecuador

**Keywords:** antibiotic resistance, enterobacterales, escherichia coli, street food, food microbiology

## Abstract

Foodborne pathogens represent a significant cause of negative impacts on human health and the economy worldwide. Unfortunately, information about epidemiological insights in Latin American countries is scarce. The consumption of ready-to-eat street food in Ecuador is extensive, and information about the presence of foodborne pathogens, their virulence factors, and antimicrobial resistance is negligible. This data includes the occurrence, phenotypic antibiotic resistance profiles, and antibiotic resistance genes of Enterobacterales isolated from ready-to-eat street food in Ambato, central Ecuador during 2020 and 2021. The most common genera detected were *Escherichia coli*, *Klebsiella spp.*, and *Cronobacter spp*. Agar disk diffusion assays were performed to determine their phenotypic resistance. The presence of antibiotic resistance genes conferring resistance against colistin, β-Lactams, aminoglycosides, tetracyclines, sulfonamides, fluoroquinolones, and amphenicols was detected via polymerase chain reaction (PCR) amplification.

## Introduction

This data contributes information about the antibiotic resistance profiles of Enterobacterales strains isolated from street food that will facilitate pathogen surveillance in Ecuador and Latin America. This data is useful for the scientific community to determine the presence of pathogenic *Escherichia coli* isolates and antibiotic resistance genes, including mobile colistin resistance genes, carbapenemases, quinolone resistance genes, and extended-spectrum β-lactamases present on Enterobacterales strains isolated from street food. Researchers and policymakers involved with the work related to the One Health initiative could also benefit from this data for retrospective and comparative analysis or epidemiological surveillance projects. ^1^^,^^2^

## Methods

### Enterobacterales strains

Ready-to-eat street food was obtained in the streets of the city of Ambato, Ecuador, and processed the same day. A sharp sterile blade was used to cut the samples on sterile surfaces. 10 g of each sample was placed in sterile brain heart infusion broth (BHIB) (Merck, Darmstadt, Germany) in 90 ml, shaken on a rotator for 8-10 min, and incubated for 24 h at 37 °C. A large amount of broth was inoculated on MacConkey agar plates (Merck, Darmstadt, Germany), Cromocult Coliforms Agar (Merck KGaA, Darmstadt, Germany), and CHROMagar mSuperCARBA were incubated overnight at 37 °C under aerobic conditions. Further purification was performed on Macconkey agar.

The isolates were amplified by polymerase chain reaction (PCR), analysed using agarose gel electrophoresis and visualised with Sybr Safe DNA Gel Stain. ^3^ For the identification of the isolated Enterobacterales, biochemical tests such as catalase, oxidase, TSI agar, Simmons citrate, lactose test, indole production, urea agar, methyl red test, and Voges-Proskauer were carried out and their interpretation was performed based on Bergey's manual. ^4^ Additionally, the software for Automated Biometric Identification Systems (ABIS) was used to confirm the biochemical identification results.

### Phenotypic antibiotic resistance profiles

Agar disk diffusion assays (Thermo Scientific Oxoid and Bioanalyse) on Mueller-Hinton Agar (Thermo Scientific Oxoid) were performed. Antibiograms tests were based on the measured diameter of the zones of inhibition and interpreted as sensible, intermediate or resistant by referring to CLSI breakpoints. ^5^

### Detection of *E. coli* pathotypes and antibiotic resistance genes detection via PCR

The PCR test was performed according to the standardized protocol of the UTA RAM One Health research group ^6^^,^^7^: 2.5 μL of DNA from each sample and 22.5 μL of PCR mix containing 12.5 μL DreamTaq PCR Master Mix (ThermoFisher Scientific, USA), 9 μL Nuclease-free water, 0.5 μL Primer 1 and 0.5 μL Primer 2 (final concentration of primers: 0.5 μM) were mixed to run PCR. The PCR conditions are reported in Supplementary Table S4. PCR products were analyzed by 1.2% agarose gel electrophoresis stained by Sybr Safe DNA Gel Stain (ThermoFisher Scientific, USA).

**Hierarchical clustering**

Hierarchical clustering was performed using the Euclidean correlation method and clustered by affinity. ^2^ The MeV Multiexperiment Viewer software version 4.8.1 was used in this study.

### Dataset validation

**Figure 1.**
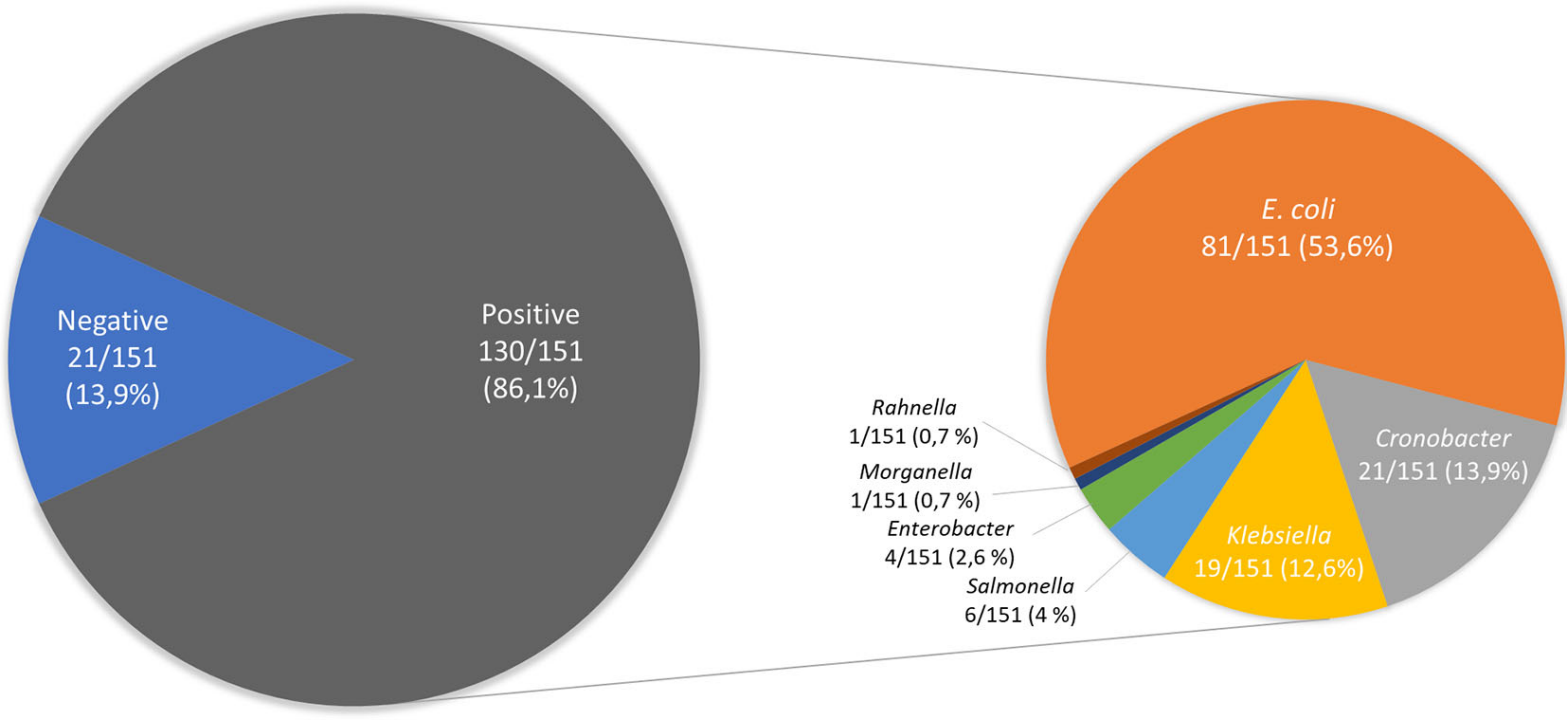
Occurrence of Enterobacterales on 151 samples of ready-to-eat street food in Ambato, Ecuador.

The data presented show the frequency of isolation of *Enterobacterales* in 151 samples of ready-to-eat street food in Ambato, Ecuador ( Figure 1). The specific characteristics (date of sampling, type of street food, location) of the samples were reported in Supplementary Table S1. A total of 145 isolates were analyzed, and the results of the biochemical tests were reported in Supplementary Table S2. Among them, 86 isolates corresponded to *E. coli* and 59 isolates to other Enterobacterales.

**Figure 2.**
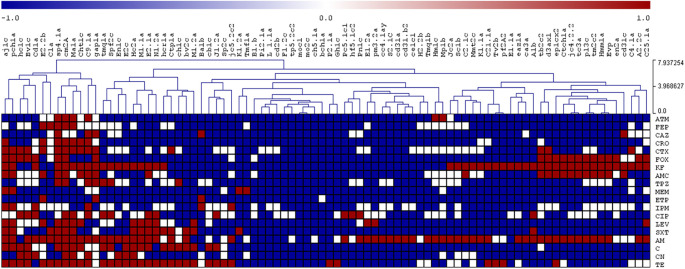
Profiles of antibiotic resistance and hierarchical tree of *E. coli* isolates. Red: resistant, White: intermediate, Blue: sensitive.

**Figure 3.**
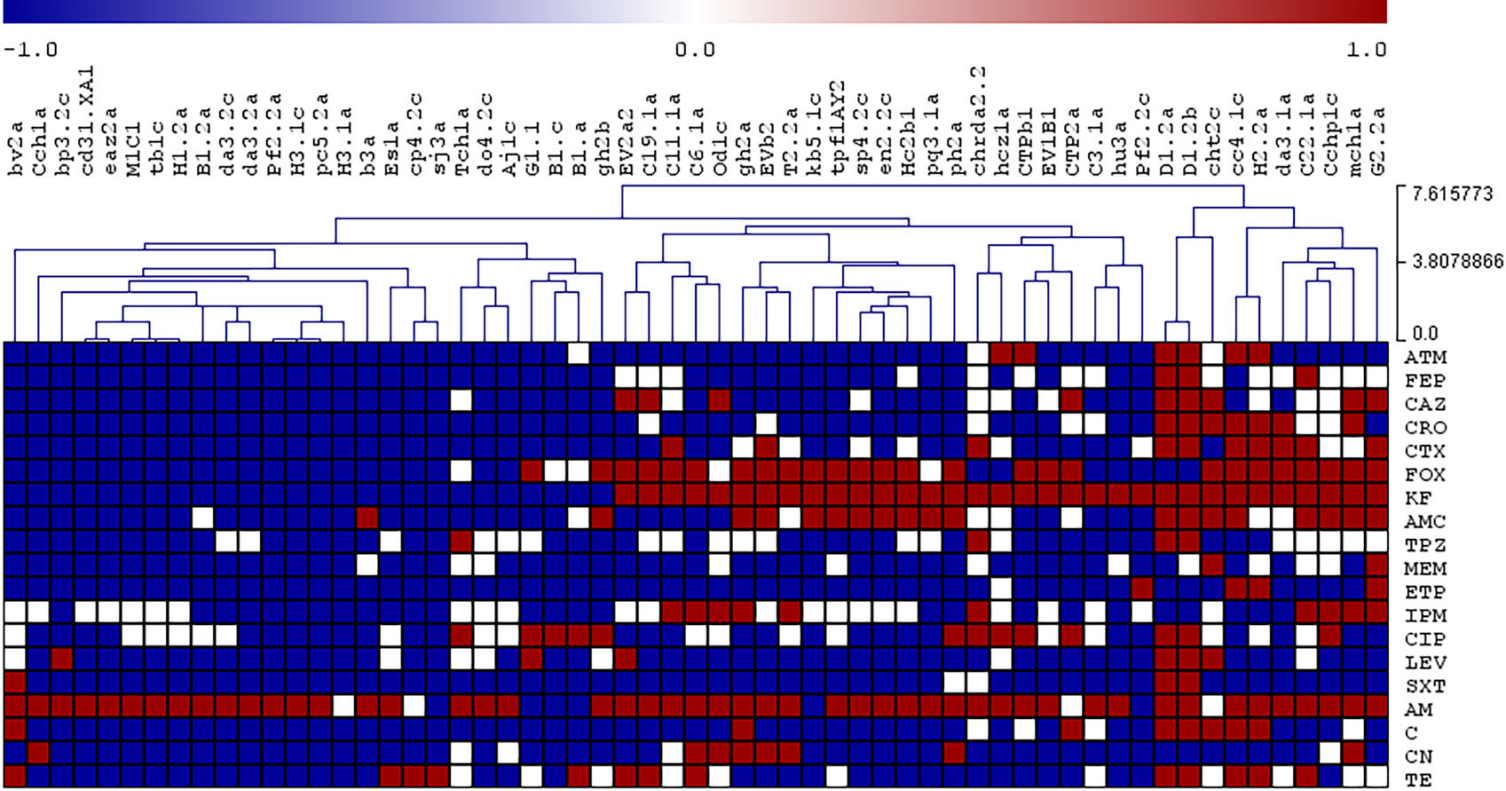
Profiles of antibiotic resistance and a hierarchical tree of other *Enterobacterales* isolates. Red: resistant, White: intermediate, Blue: sensitive. Abbreviations: TE: Tetracycline 30 μg, AM: Ampicilin 10 μg, KF: cephalotin 30 μg, C: chloramphenicol 30 μg, CIP: Ciprofloxacin 5 μg, CTX: Cefatoxime 30 μg, LEV: Levofloxacin 5 μg, FOX: Cefoxitin 30 μg, STX: Trimethoprim/sulphamethoxazole 25 μg, AMC: Amoxicyllin/ClavulanicAcid 30 μg, CN: Gentamicin 10 μg, CRO: Ceftriaxione 30 μg, FEP: Cefepime 30 μg, ATM: Aztreonam 30 μg, IPM: Imipenem 10 μg, TPZ: Piperacillin/Tazobactam 110 μg, ETP: Ertapenem 10 μg, MEM: Meropenem 10 μg, CAZ: Ceftazidime 30 μg.

To visualize the relative similarity of the antimicrobial resistance patterns of the isolates, a hierarchical cluster analysis was performed using the results of the antibiograms, where the phenotypes ‘resistant’, ‘intermediate’, and ‘susceptible’ were observed as red, white, and blue colors respectively. Dendrograms and clustered data were assembled using the complete linkage method through Pearson correlation and sample leaf organization. ^7^ For this purpose, the MeV Multiexperiment Viewer software version 4.8.1 was used. ^8^ Figures 2 and 3 represent the resistance profiles and the hierarchical clustering of *E. coli* and the rest of Enterobacterales, respectively. The complete information is shown in Supplementary Table S3.

The presence of diarrheagenic *E. coli* pathotypes present in ready-to-eat food was assessed in this study through the analysis of virulence genes related to the pathotypes. Only one isolate (C2.1c) was positive for the eae gene, suggesting the potential presence of enteropathogenic *E. coli* (EPEC) or enterohemorrhagic *E. coli* (EHEC). The β-lactamase resistance genes of Enterobacterales isolated in this study are reported in Table 1. Mobile colistin resistance genes or quinolone resistance genes were not found in the Enterobacterales isolates. The complete information about virulence genes and antibiotic resistance genes are available in Supplementary Table S5. The information about primers and PCR conditions were shown in Supplementary Table S4. The gel electrophoresis images are available at Supplementary figure S6. The disk difussion assays figures were shown at Supplementary figure S7.

**Table 1.** Beta-lactamase resistance genes of *Enterobacterales* isolated from ready-to-eat food.

Ready-to-eat food samples	Bacteria	Sample ID	Date	Location market streets	Beta-lactamases	
					*bla* *tem*	*bla* *cmy*
Cow stomach stew	*E. coli*	M1.1a	14/12/2020	Mayorista	+	-
Cane juice	*E. coli*	Jc5.2c1	08/03/2021	Modelo	+	-
Lupine ceviche	*E. coli*	ch5.1c2	08/03/2021	Modelo	+	-
Cow stomach stew	*E. coli*	Gap1a	20/04/2021	Sur	-	+
Boiled beans, ulluco and pork rind	*E. coli*	Hmm1a	10/05/2021	Artesanal	-	+
Chilli sauce	*Klebsiella* spp	D1.2a	07/12/2020	Mayorista	+	-
Sweet meringue (espumilla)	*Salmonella* spp	E2.2b	19/01/2021	Primera de Mayo	+	-
Salad from street food	*E. coli*	N1.2a	20/12/2020	Mayorista	+	-
Total *Enterobacterales* isolated					6(145)	2(145)

## Data availability

Figshare project: https://figshare.com/projects/Data_on_antibiograms_and_resistance_genes_of_Enterobacterales_isolated_from_Ready-to-eat_street_food_of_Ambato_Ecuador/137014

This collection contains the following underlying data:

Figure 1. Occurrence of Enterobacterales on 151 samples of ready-to-eat street foods in Ambato, Ecuador. figshare. Figure. https://doi.org/10.6084/m9.figshare.19579087.v1 ^9^

Table 1. Beta-lactamase resistance genes of Enterobacterales isolated from ready-to-eat food. figshare. Dataset. https://doi.org/10.6084/m9.figshare.19579099.v1 ^10^

Figure 2 and 3. Antibiotic resistance profiles and hierarchical trees of Enterobacterales isolated from ready-to-eat street food in Ambato, Ecuador. figshare. Dataset. https://doi.org/10.6084/m9.figshare.19579267.v1 ^11^

### Extended data

This collection contains the following extended data:

Supplementary table S1. Characteristics (Sample type, date, treatment type, location, coordinates) of the ready-to-eat food samples. figshare. Dataset. https://doi.org/10.6084/m9.figshare.19579108.v1 ^12^

Supplementary table S2. Biochemical tests performed on Enterobacterales isolates from Ready-to-eat Street Food in Ambato, Ecuador. figshare. Dataset. https://doi.org/10.6084/m9.figshare.19579177.v1 ^13^

Supplementary table S3. Antibiogram of Enterobacterales isolated from ready-to-eat Street food of Ambato, Ecuador. figshare. Dataset. https://doi.org/10.6084/m9.figshare.19579189.v1 ^14^

Supplementary table S4. Primers used in this study and PCR conditions. figshare. Dataset. https://doi.org/10.6084/m9.figshare.19579198.v1 ^15^

Supplementary table S5. Antibiotic resistance genes and virulence genes harbored by Enterobacterales isolates from ready-to-eat street food in Ecuador. figshare. Dataset. https://doi.org/10.6084/m9.figshare.19579207.v1 ^16^

Supplementary figure S6. PCR results (positive electrophoresis images). figshare. Figure. https://doi.org/10.6084/m9.figshare.19729618.v1 ^17^

Supplementary figure S7. Disk diffusion assay images-Antibiotic resistance evaluation of Enterobacterales isolated from ready-to-eat street food of Ambato, Ecuador. figshare. Figure. https://doi.org/10.6084/m9.figshare.19729630.v1 ^18^

Data are available under the terms of the Creative Commons Attribution 4.0 International license (CC-BY 4.0).
