# Supplementary material for: Data on antibiograms and resistance genes of Enterobacterales isolated from ready-to-eat street food of Ambato, Ecuador
Source: F1000Res. 2022 Jun 17;11:669. [Version 1] doi: 10.12688/f1000research.117116.1 (PMC9257263; doi:10.12688/f1000research.117116.1)
Supplement: Peer review history

# Peer review: Reviewer response for version 1

**DOI:** 10.5256/f1000research.128952.r141527

**Published:** 2022-07-19

**Authors:** Erika A. Rodriguez

**Affiliations:** 1 Bacterial Molecular Epidemiology Line, Research Group in Basic and Applied Microbiology (MICROBA), School of Microbiology, University of Antioquia, Medellín, Colombia

The data note by Tubón J et al. aims to give information about the antibiotic resistance profiles of Enterobacterales strains isolated from street food in Ecuador. The work is relevant because, usually in Latin American countries, street food consumption is typical, it has little sanitary regulation, and in some towns, potable water access is limited, which could favor the presence of resistant bacteria in foods. On the other hand, the research gives information about antibiotic resistance in settings different from hospitals and contributes information from Latin American countries where the data is limited. Another relevant point is that the work detects antibiotic resistance Enterobacterales strains in cooked food differently from other studies on lettuce and raw vegetables.

 The manuscript is well structured and written. The protocols are appropriate and provide acceptable methods and details to allow replication.

 I have provided comments/suggestions as follows:

- I suggest to future reports, the author gives information about the bacteria typification, such as ERIC, PFGE, MLST, or WGS. This information could be help to understand what clones to type of strains are circulating in the region.
- What is the resistance behavior in the Ambato hospitals where the project was carried out? The results could help to understand the resistance problem in the city? 
- Any limitations related to this manuscript and/or methods you want to bring up to the reader?
- In the profiles of antibiotic resistance, there are Enterobacterales isolates resistant to carbapenems, and you did not detect carbapenemases. Could you give information about the reason to this resistance, for example, intrinsic mechanisms or other beta-lactamases not detected?

Are sufficient details of methods and materials provided to allow replication by others?

Yes

Is the rationale for creating the dataset(s) clearly described?

Yes

Are the datasets clearly presented in a useable and accessible format?

Yes

Are the protocols appropriate and is the work technically sound?

Yes

Reviewer Expertise:

Bacterial Antibiotic Resistance, Molecular Microbiology, Molecular Epidemiology, Medical Microbiology.

I confirm that I have read this submission and believe that I have an appropriate level of expertise to confirm that it is of an acceptable scientific standard.

# Peer review: Reviewer response for version 1

**DOI:** 10.5256/f1000research.128952.r141528

**Published:** 2022-07-05

**Authors:** Edgar Gonzalez-Villalobos

**Affiliations:** 1 Catalan Institute for Water Research (ICRA), Girona, Spain

In Latin American countries the consumption of street food represents in the majority the alimentary base, due to the conditions that present the most countries belonging to Latin American, for this reason, the information generated by Turbón et al., could facilitate the epidemiology surveillance and allow action before the emergence of foodborne pathogens outbreak.

 The authors carry out a complete characterization of a significant number of strains (n=151), highlighting the distribution of pathogens, antibiotic resistance profiles, presence of antibiotic resistance genes.

 I suggest reviewing next points:

1. The sentence that refers to " *The isolates were amplified by polymerase chain reaction (PCR)*" confuses a bit since later it is specified that the identification is carried out with conventional biochemicals, just to clarify well, why PCR was used in this section.
2. Add information about how to DNA was obtained for PCR assays.
3. Was there any pathogen that predominated in the sampling areas? If so, the results could show a strain that is already distributed in the community with outbreak potential.

Both articles (that are referenced below) are related with the importance of a good epidemiological surveillance system in a possible outbreak of foodborne pathogens. These are closely related to the study presented by the authors, where they carry out the isolation and complete characterization, anticipating of the emergence of foodborne pathogens in an area such as Latin America where epidemiological data usually are limited.

Are sufficient details of methods and materials provided to allow replication by others?

Yes

Is the rationale for creating the dataset(s) clearly described?

Yes

Are the datasets clearly presented in a useable and accessible format?

Yes

Are the protocols appropriate and is the work technically sound?

Yes

Reviewer Expertise:

antibiotic resistance, microbiology, phages

I confirm that I have read this submission and believe that I have an appropriate level of expertise to confirm that it is of an acceptable scientific standard.

## References

[1] : PulseNet Latin America and the Caribbean Network: Present and Future. *Foodborne Pathog Dis* .16(7) : 10.1089/fpd.2018.2587 489-497 10.1089/fpd.2018.2587 31081688PMC6653793

[2] : Using Market Availability Data to Support Foodborne Disease Outbreak Investigations. *American Journal of Public Health* .2020;110(3) : 10.2105/AJPH.2019.305536 278-280 10.2105/AJPH.2019.305536 PMC700293332023105

# Peer review: Reviewer response for version 1

**DOI:** 10.5256/f1000research.128952.r141529

**Published:** 2022-06-23

**Authors:** Laura Sala-Comorera

**Affiliations:** 1 UCD School of Biomolecular and Biomedical Science, UCD Earth Institute, Dublin, Ireland; 2 UCD Conway Institute, University College Dublin, Dublin, Ireland

In this data note article, Turbón and colleagues examined the antibiograms and resistance genes profiles of Enterobacterales order strains isolated in ready-to-eat street food in Ecuador. A larger number of samples and types of food have been selected, resulting in a solid database of Enterobacterales strains (n=151).

 I suggested a few points to be clarified.

- Methods
  - Enterobacterales strains- Section
    - “A larger amount of broth”. The authors can specify the volume used for the analysis.
    - Add the reference for the “software for Automated Biometric Identification Systems (ABIS)”.
  - Phenotypic antibiotic resistance profiles – Section. 
    - Include the full reference for these companies (Thermo Scientific Oxoid and Bioanalyse) following the same style as the previous ones.
  - Detection of *E. coli* pathotypes and antibiotic resistance genes detection via PCR - Section.
    - Include the method/kid used for the DNA extraction.

Are sufficient details of methods and materials provided to allow replication by others?

Yes

Is the rationale for creating the dataset(s) clearly described?

Yes

Are the datasets clearly presented in a useable and accessible format?

Yes

Are the protocols appropriate and is the work technically sound?

Yes

Reviewer Expertise:

water microbiology, food microbiology, microbial source tracking

I confirm that I have read this submission and believe that I have an appropriate level of expertise to confirm that it is of an acceptable scientific standard.
